# Synthesis of Carbon-Encapsulated Magnetic Iron Oxide Nanocomposites for Bioapplication

**DOI:** 10.1155/2022/3302082

**Published:** 2022-09-20

**Authors:** Wubshet Mekonnen Girma

**Affiliations:** Department of Chemistry, Wollo University, Dessie, Ethiopia

## Abstract

Carbon-encapsulated Fe_3_O_4_ nanoparticles (NPs) were successfully synthesized from a single precursor using one-step solvothermal methods. X-ray diffraction and transmission electron microscopy were used to characterize the as-prepared NPs, and UV-visible absorbance spectroscopy was used to check their optical properties. The morphological results revealed that Fe_3_O_4_@C, quasi-spherical Fe_3_O_4_ particles encapsulated by carbon. In addition, the carbon-encapsulated Fe_3_O_4_ NPs were conjugated with folic acid (FA) to be used as biomarkers in the diagnosis and treatment of tumour cells. Fourier transform infrared spectroscopy and UV-visible spectroscopic techniques were used to confirm the conjugation process.

## 1. Introduction

Nanoparticles (NPs) that combine both magnetic and optical properties have attracted attention owing to their wide range of biomedical applications. Magnetic NPs can be tailored as a contrast agent in magnetic resonance imaging (MRI), for separation, detection, targeting, hyperthermia, and photothermal therapy. Magnetic nanocrystalline materials have the disadvantage of rapid degradation/aggregation in the physiological environments of the tumour as well as aqueous solubility due to very high surface area to volume ratio and higher reactivity. This tends to restrict their potential application and investigation of their properties [1]. To overcome this problem encapsulation has been done by different scholars to protect aggregations and exploit their properties to apply for different applications [2, 3]. A number of magnetic NP formulations are currently being developed to meet specific needs; however, little effort has been made to develop a universal combined formulation [4] for cancer applications. As a result, developing a multifunctional magnetic NP formulation that does not compromise fundamental properties is highly desirable. Such formulations have recently been developed to gain various biological functions [5]. These formulations can be used for not only drug delivery but also MRI visible targeting [6], magnetically targeted photodynamic therapy [7], targeted thermosensitive chemotherapy [8], and luminescence/near-IR/multi-model imaging [9].

Among different magnetic materials, Fe_3_O_4_ is widely studied magnetic nanocluster and applicable for hyperthermia, MRI, cancer staining, hydrogen storage and supercapacitors, soil remediation, and water treatments. However, Fe_3_O_4_ suffer from two major issues such as rapid agglomeration, oxidation into the physiological environment of the tumours due to large surface area, chemical reactivity, and high surface energy, thus resulting in a loss of magnetism [10, 11]. Therefore, encapsulation/surface modification plays a crucial role in its structure since it can protect the magnetic core from oxidation, thermal and temporary degradation [12]. However, Fe_3_O_4_ coated by polymers, nanogels, micelles, and microcapsules exhibited reduction of magnetic property, increments of particle size, and lower efficiency during drug loading. In addition, it affects the solubility, leads to aggregation, and further affects biocompatibility in living cells in some of these complexes. Therefore, the carbon encapsulation adds additional features to the magnetic NPs such as easy functionalization, electrical properties, as well as catalytic activities. It also helps to stabilize at physiological and high temperature environment to make it biocompatible in medical applications. Most recent studies showed that folic acid (FA) modified Fe_3_O_4_ NPs are applied for cervical cancer diagnosis [13]. Since recent studies displayed that specific biomarkers on the surface of NPs lead to efficient diagnosis and treatments of cancer [14–16].

In this work, we report a single and rapid synthesis protocol by using the hydrothermal approach from a single precursor, carbon-encapsulated Fe_3_O_4_ NPs are formed via oxidation of Fe^2+^ using H_2_O_2_, by decomposition of ferrocene. The obtained NPs have good magnetic properties with carbon encapsulation, allowing us to conjugate with folic acid and use them in biomedical applications such as diagnosis, therapeutic, environmental remediation, catalysis, energy storage materials, and magnetic devices. The advantages of our formulation are good magnetic properties, good stability, and easy functionalization due to carbon encapsulation. Furthermore, the biocompatibility of the synthesized magnetic material has displayed a minimal toxicity. Therefore, it could have promising applications in biomedical fields.

## 2. Materials and Methods

### 2.1. Chemicals

Ferrocene (Fe(C_5_H_5_)_2_, ≥98%), hydrogen peroxide (H_2_O_2_, 30%), acetone (C_3_H_6_O, ≥99%), and 3-(4, 5-dimethylthiazol-2-yl)-2, 5-diphenyltetrazolium bromide (MTT, 97.5%) were obtained from Sigma-Aldrich. Folic acid (>98%) was obtained from TCI. N-hydroxy sulfosuccinimide sodium salt (Sulfo-NHS, 97%), ethyl (dimethyl aminopropyl) carbodiimide (EDC, 99%), and N-hydroxysulfosuccinimide sodium salt (sulfo-NHS, 97%) were obtained from Alfa-Aesar.

### 2.2. Synthesis of Fe_3_O_4_@C Core/Shell NPs

The synthesis protocol was done with small modifications from the literature [10]. 0.2 g of ferrocene was dissolved in 60 mL of acetone and subjected to sonication for half an hour. 30% of H_2_O_2_ was slowly added to the solution with continuous stirring for half hour. Afterward, the solution was transferred to Teflon-lined stainless autoclave and allowed to proceed for 16 h at 180°C. Then, the autoclave was allowed to cool at room temperature. The product was collected after intense sonication by using magnet and washed with acetone to remove unreacted ferrocene. Finally, the obtained Fe_3_O_4_@C NPs were dried at 40°C in vacuum.

### 2.3. Conjugation of Fe_3_O_4_@C with Folic Acid

To activate the carboxylic group of Fe_3_O_4_@C NPs, EDC (10 mg) and sulfo-NHS (10 mg) in 1 mL of MES buffer were added to 2 mL of Fe_3_O_4_@C NPs solution (20 mg/mL) and then stirred in the dark at room temperature for 30 min. Subsequently, 1 mL of MES solution containing folic acid (2.26 × 10^−3^ mmol) was added to the activated Fe_3_O_4_@C NPs solution, and the mixture was stirred gently at room temperature in the dark. After 16 h, a magnet was used to separate the final product (Fe_3_O_4_@C-FA).

### 2.4. Cell Culture and Cell Viability Test

Human cervical cancer cells (HeLa) were cultivated in Dulbecco's modified eagle's medium (DMEM, Hyclone), added with 1% nonessential amino acids, 1% sodium pyruvate, 1% L-glutamine, 1% antibiotic antimycotic formulation, and 10% fetal bovine serum at 37°C, in a humidified 5% CO_2_ atmosphere.

Fe_3_O_4_@C-FA *in vitro* cytotoxicity was assessed using the MTT assay. Cells (∼5 × 104/well) were cultured in a 24-well plate for 24 h. Afterward, the medium was washed three times and changed with 200 *μ*L fresh culture medium contain various concentrations (0, 25, 50, 100, 200, 500, and 1000 *μ*g/mL) of Fe_3_O_4_@C-FA and cultured for additional 24 h. Then, the cultured cells washed with PBS three times and replaced with fresh medium followed by adding 1 mL of MTT aqueous solution (500 *μ*g/mL) was added to each well, and the cells were incubated at 37°C in a 5% CO_2_ humidified incubator for another 4 h. Subsequently, the medium was removed, and cells were dispersed into 1 mL of dimethyl sulfoxide/well and incubated for 15 min. Finally, the optical density of the cell suspension was measured at 570 nm using a BiotekPowerwave XS plate reader. The experiment was done three times under the same conditions. The cell viability was estimated with the following equation:

(1) $$\begin{array}{c} \text{cell viability}\left(\%\right)=\frac{\text{optical density of treated sample}}{\text{optical density of control sample}}\times 100\%. \end{array}$$

### 2.5. Characterization

The morphology was characterized using transmission electron microscopy (TEM) and FEI Tecnai G2 F20 microscope (Philips, Amsterdam, Holland) with an accelerating voltage of 200 kV. X-ray diffraction (XRD) patterns were obtained using a Bruker D8 Discover X-ray diffractometer. UV-visible absorbance spectra were acquired on Jasco V-630 spectrophotometers. Fourier transform infrared (FT-IR) spectra were obtained by Nicolet 5700 FT-IR spectrometer.

## 3. Results and Discussion

### 3.1. Synthesis and Characterizations of Fe_3_O_4_@C

**Scheme 1:**
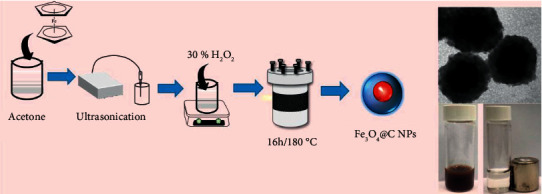
The schematic representations of one-step fabrications of Fe_3_O_4_@C from a single precursor.

As shown in Scheme 1, Ferrocene has been employed as an adaptable precursor to synthesize carbon-encapsulated Fe_3_O_4_ NPs since it can offer both iron and carbon sources [17, 18]. Acetone were used as a synthesis solvent and ultrasonication were used to homogenise ferrocene. Hydrogen peroxide were added to oxidize ferrocene into Fe_3_O_4_@C. Precursor thermally decompose and monomers accumulate in the solution during the heat treatment. Burst nucleation occurred when the monomer concentration exceeded a critical level for nucleation, followed by the growth of Fe_3_O_4_@C NPs.

**Figure 1:**
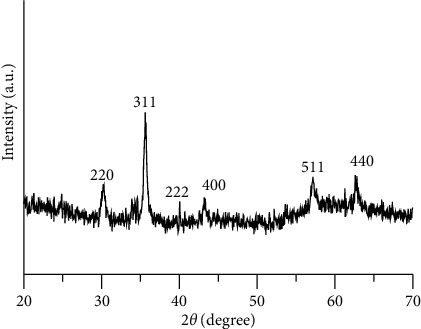
The XRD patterns of as-prepared Fe_3_O_4_@C.

Powder X-ray diffraction technique was applied to identify the crystalline structure of the as-prepared Fe_3_O_4_@C NPs.  Figure 1 clearly displays five diffraction peaks that match with (220), (311), (222), (400), (511), and (440) lattice planes of Fe_3_O_4_ (JCPDS 19-0629). The higher diffraction intensity implies that higher crystalline magnetic Fe_3_O_4_ particles synthesized by the solvothermal method. It should be noted that due to the thin layer of the carbon shell on Fe_3_O_4_ nanospheres, no peaks for amorphous carbon were observed, suggesting the structure of carbon is amorphous in the sample. There were no other impurity peaks detected, indicating the high purity of the synthesized products.

**Figure 2:**
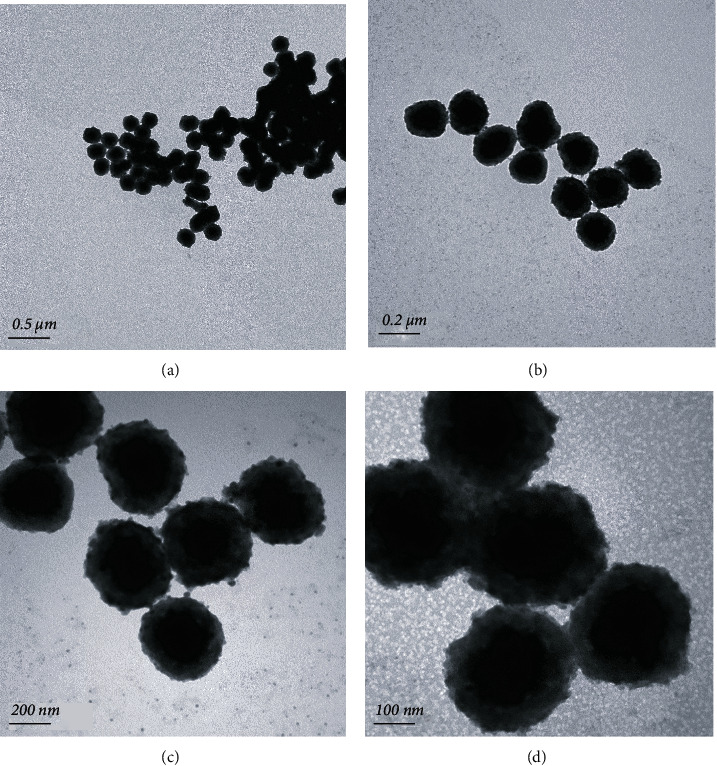
(a–d) The TEM images of the as-prepared Fe_3_O_4_ NPs at different scale bars.

Figure 2 displays the typical TEM images of the as-synthesized Fe_3_O_4_@C NPs to see the morphology and microstructures. At first glance, it is clear from Figures 2(a) and 2(b) that the products have a sphere-shaped morphology. It should be noted that the majority of Fe_3_O_4_ particles were coated in carbon close scrutiny (Figures 2(c) and 2(d)) demonstrates that the thickness of the carbon layer on the surface of Fe_3_O_4_ was very thin and uneven. The dimensions of the Fe_3_O_4_ core were significantly larger than the carbon shell layer. Generally, well-dispersed NPs with Fe_3_O_4_ encapsulated by carbon matrix from a single precursor. Furthermore, agglomeration of NPs was not seen, this might be due to successful encapsulation of the NPs.

**Figure 3:**
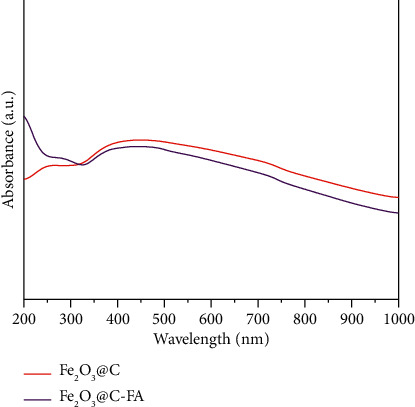
The absorbance spectra of Fe_3_O_4_@C and Fe_3_O_4_@C-FA.

The optical properties of the as-prepared NPs were studied using absorbance spectroscopy. Both Fe_3_O_4_@C and Fe_3_O_4_@C-FA showed absorbance profile in UV and visible spectral ranges 200–1000 nm with long tail in longer wave length as shown in Figure 3. The absorption profiles of Fe_3_O_4_@C-FA showed similar profile as its original NPs except intensity change.

**Figure 4:**
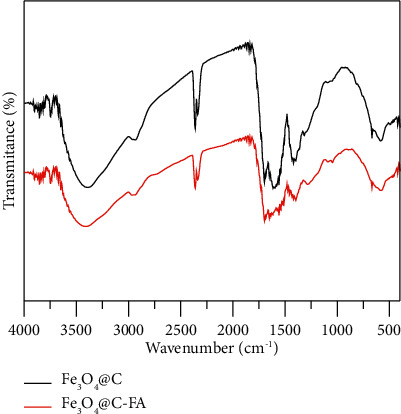
The FT-IR spectra of Fe_3_O_4_@C and Fe_3_O_4_@C-FA.

As depicted in Figure 4, FT-IR spectra were chosen to identify the functional groups in each conjugation steps. A broad peak appearing above 3300 cm^−1^ were assigned to the stretching vibrations of-OH and -NH_2_ groups. The stretching peaks appearing around 2953 cm^−1^ were assigned to C-H in both Fe_3_O_4_@C and Fe_3_O_4_@C-FA. The stretching peaks appearing around 2343 cm^−1^ were due to C=O=C searching frequencies. The stretching peaks appearing around 1530–1680 cm^−1^ were assigned to C=C searching frequencies. After conjugation, thus C=C peaks showed shift. A medium band appearing around 1424 cm^−1^ in Fe_3_O_4_@C NPs was assigned C-H bending vibrations. This peak in Fe_3_O_4_@C-FA shifted to 1410 cm^−1^ with intensity decrement which reveled successful conjugations.

**Figure 5:**
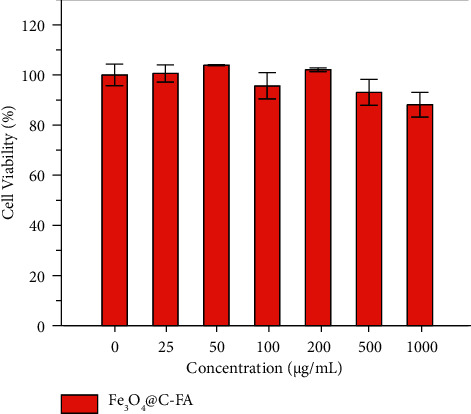
The MTT assay of HeLa cells cell viable after incubations of different concentrations (0, 25, 50, 100, 200, 500, and 1000 *μ*g/mL) of Fe_3_O_4_@C-FA at 37°C for 48 h. Error bars indicate the mean ± standard deviations (*n* = 3).

The *in vitro* cytotoxicity of the Fe_3_O_4_@C-FA was investigated by the MTT assay against HeLa cells. As displayed in Figure 5, the viability of cells was greater than 95% after treatments of cells for 48 h at different concentrations of Fe_3_O_4_@C-FA (0, 25, 50, 100, 200, 500, and 1000 *μ*g/mL). Accordingly, Fe_3_O_4_@C-FA showed lower cytotoxicity, which makes it competent for future clinical applications. Thus, the results confirmed that Fe_3_O_4_@C-FA was highly biocompatible with low toxicity to serve as bioapplications.

## 4. Conclusions

In conclusion, a simple one-pot solvothermal synthesis method using a single precursor was developed to serve as a biomedical probe in the diagnosis and therapy. The entire synthesis protocol for carbon-encapsulated iron oxide leads to magnetic, stable, and biocompatible NPs and is fast, reproducible, and scalable. The TEM microstructures revealed that the Fe_3_O_4_ NPs are carbon encapsulated, and thus free of aggregation, which is a common problem in most magnetic nanoparticles. The cytotoxicity study confirmed the low toxicity for biological applications.

## Data Availability

All data are included within the article.
